# Biomolecule Profiles in Inedible Wild Mushrooms with Antioxidant Value

**DOI:** 10.3390/molecules16064328

**Published:** 2011-05-25

**Authors:** Filipa S. Reis, Eliana Pereira, Lillian Barros, Maria João Sousa, Anabela Martins, Isabel C.F.R. Ferreira

**Affiliations:** 1 Mountain Research Centre (CIMO), Instituto Politécnico de Bragança, Campus de Santa Apolónia, Apartado 1172, 5301-855 Bragança, Portugal; Email: filipa.reis@gmail.com (F.S.R.); eliana.pereira_19@hotmail.com (E.P.); lillian@ipb.pt (L.B.); joaos@ipb.pt (M.J.S.); 2 Escola Superior Agrária, Instituto Politécnico de Bragança, Campus de Santa Apolónia, Apartado 1172, 5301-854 Bragança, Portugal; Email: amartins@ipb.pt (A.M.)

**Keywords:** wild mushrooms, biomolecules, antioxidants, antioxidant activity

## Abstract

The use of natural products isolated from mushrooms, included inedible species, against infection, cancer diseases and other oxidative-stress related diseases is one of the cornerstones of modern medicine. In the present work, the antioxidant molecule profiles of inedible mushroom species were evaluated and compared with those of edible species. The order of antioxidant abundance found in inedible wild mushrooms was: phenolics > flavonoids > ascorbic acid > tocopherols > carotenoids, similar to that of edible species. Furthermore the same energetic biomolecules were found including the disaccharide trehalose, the monosaccharide alcohol derivative mannitol and the fatty acids palmitic, oleic and linoleic acids. *Fomitopsis pinicola* revealed a very high phenolics concentration (388 mg GAE/g extract) and powerful antioxidant properties, mainly reducing power (EC_50_ value 60 μg/mL similar to the standard Trolox^®^). It could find applications in the prevention of free radical-related diseases as a source of bioactive compounds.

## 1. Introduction

More than 3000 mushrooms are said to be ‘prime edible species’, of which only some 100 are cultivated commercially, and only ten of those on an industrial scale. Their global economic value is nevertheless now staggering, and a prime reason for the rise in consumption is a combination of their value as a food as well as their medicinal and nutraceutical (*i.e.*, dietary supplement) values [1]. This number increases dramatically if inedible species are considered. A lot of inedible mushrooms show bitter and pungent taste, and especially those belonging to the Polyporaceae have been used as medicinal drugs in China from ancient times [2]. Even edible species of potent medicinal mushrooms such as *Ganoderma lucidum*, *Trametes versicolor* and *Inonotus obliquus* are very bitter and/or hard to eat and are thus used in the form of an extract, tea or powder [3,4].

The use of natural products isolated from mushrooms against infectious and neoplastic diseases is one of the cornerstones of modern medicine. Bioactive molecules have been isolated not only from edible (e.g., *Ganoderma applanatum* and *Agaricus* spp.) but also from inedible species such as the ones belonging to the Scutigeraceae, Polyporaceae, Xylariaceae, Thelephoraceae and Paxillaceae families [2]. Mushrooms’ reported bioactivities include antibacterial, antifungal, antioxidant, antiviral, anti-tumor, cytostatic, immunosuppressive, antiallergic, antiatherogenic hypoglycemic, anti-inflammatory and hepatoprotective activities [5,6]. 

The most widely distributed molecules with antitumor properties in mushrooms are sesquiterpenes, triterpenoids, glucans and glycoproteins [7]. Other important molecules are those with antioxidant properties as they can help the endogenous defence system against oxidative stress caused by the excess of reactive oxygen and nitrogen species (ROS and RNS). The non-controlled production of those species has been related to more than one hundred diseases, including several kinds of cancer, diabetes, cirrhoses, cardiovascular diseases, neurological disorders, as also to the aging process [8]. Wild mushrooms contain different antioxidants such as phenolic compounds, tocopherols, ascorbic acid, and carotenoids which could be extracted for the purpose of being used as functional ingredients namely against chronic diseases related to oxidative stress [9]. 

In the present work, we aimed to evaluate the antioxidant molecule profiles of inedible (non-palatable) mushroom species and to compare them with edible species that we have extensively studied [10,11,12].

## 2. Results and Discussion

### 2.1. Biomolecules with Energetic Value

**Figure 1:**
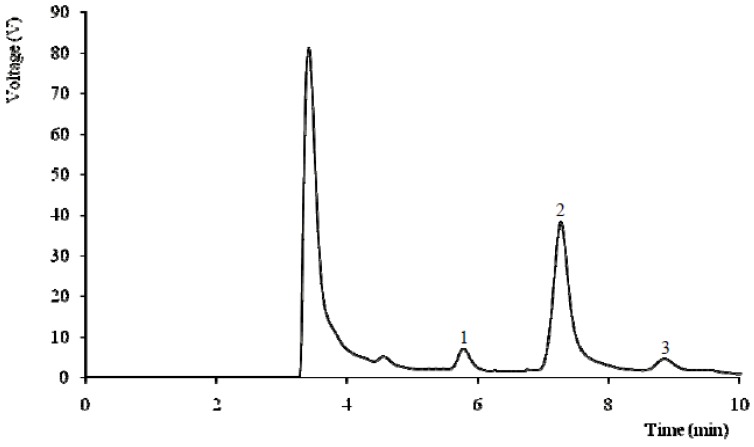
Individual sugars chromatogram of *Lentinus tigrinus*: 1-mannitol; 2-trehalose; 3-raffinose (IS).

The biomolecules with energetic value found in the studied wild mushrooms are shown in Table 1, Table 2, Table 3, Table 4. Sugars are very important in cellular energetic metabolism contributing to the support and expansion of mushrooms fruiting bodies [12]. Mannitol and trehalose were the main sugars in the studied species (Table 1). The highest levels of total sugars (50 g/100 g dw) and trehalose (41 g/100 g dw) were found in *Lentinus tigrinus*, and its individual sugar profile can be observed in Figure 1. The species where fructose was found are all mycorrhizal, which is in agreement with our previous results [11]. 

**Table 1:** Biomolecules (free sugars) with energetic value.

Species	Fructose (g/100 g dw)	Mannitol (g/100 g dw)	Trehalose (g/100 g dw)	Total Sugars (g/100 g dw)
*Amanita porphyria*	nd	2.40 ± 0.42 ef	1.41 ± 0.16 gf	3.81 ± 0.58 e
*Collybia fusipes*	nd	9.27 ± 0.76 d	21.47 ± 1.99 c	30.74 ± 2.75 c
*Fomitopsis pinicola*	nd	6.49 ± 0.44 ed	3.17 ± 0.66 f	9.66 ± 1.10 d
*Hebeloma sinapizans*	0.25 ± 0.02 a	0.64 ± 0.01 f	28.75 ± 3.38 b	29.63 ± 3.41 c
*Inocybe splendens*	nd	8.53 ± 1.42 d	6.10 ± 1.41 e	14.63 ± 2.84 d
*Lactarius hepaticus*	nd	27.39 ± 2.00 c	1.86 ± 0.45 gf	29.25 ± 2.45 c
*Lentinus tigrinus*	nd	7.05 ± 0.83 d	41.49 ± 0.89 a	48.54 ± 0.06 a
*Piptoporus betulinis*	nd	0.36 ± 0.04f	12.15 ± 0.65 d	12.51 ± 0.69 d
*Pluteus murinus*	nd	0.98 ± 0.01 f	0.17 ± 0.01 g	1.14 ± 0.00 e
*Russula emetica*	0.28 ± 0.17 a	36.98 ± 6.57 b	2.87 ± 0.27 gf	40.13 ± 6.47 b

nd- not detected. In each column different letters mean significant differences between species (*p* < 0.05).

The results of the main fatty acids found in the studied wild mushrooms, as also their saturated fatty acids (SFA), monounsaturated fatty acids (MUFA) and polyunsaturated fatty acids (PUFA) percentages are shown in Table 2. 

Up to twenty-eight fatty acids were detected in most of the samples (data not shown). The major fatty acid found was linoleic acid (C18:2n6) (prevalence of PUFA), except for *Inocybe splendens*, *Pluteus murinus* and *Russula emetic*, where oleic acid (C18:1n9) predominated, contributing to the prevalence of MUFA in those species. The studied species also revealed palmitic acid (C16:0) as a major fatty acid. *Amanita porphyria* and *Hebeloma sinapizans* gave the highest levels of PUFA (71–72%), while *Russula emetica* gave the highest levels of MUFA (66%).

Despite the abundance of palmitic acid in different organisms, linoleic and oleic acids are common in eukaryotic organisms such as fungi. Furthermore, linoleic acid is precursor of 1-octen-3-ol (“fungi alcohol”), the main aromatic component in fungi [13]. As stated by us and by other authors, UFA were higher than SFA levels [11,12,14].

**Table 2:** Biomolecules (fatty acids) with energetic value.

Species	C16:0	C18:0	C18:1n9	C18:2n6	SFA	MUFA	PUFA
*Amanita porphyria*	11.99 ± 0.42 c	2.23 ± 0.02 f	5.45 ± 0.14 h	71.22 ± 4.03 a	21.65 ± 0.26 c	6.32 ± 0.62 g	72.03 ± 3.88 a
*Collybia fusipes*	18.49 ± 0.17 a	5.22 ± 0.05 c	13.47 ± 0.01 f	55.30 ± 0.11 d	30.05 ± 0.16 a	14.08 ± 0.01 e	55.87 ± 0.14 d
*Fomitopsis pinicola*	11.81 ± 0.04 c	3.09 ± 0.08 e	16.17 ± 0.74 e	65.07 ± 0.57 b	18.15 ± 0.17 e	16.49 ± 0.74 e	65.36 ± 0.57 b
*Hebeloma sinapizans*	14.61 ± 0.14 b	3.11 ± 0.04 e	7.92 ± 0.69 g	69.89 ± 0.20 a	19.87 ± 0.23 d	8.93 ± 0.60 f	71.20 ± 0.37 a
*Inocybe splendens*	10.27 ± 0.02 d	2.20 ± 0.01 f	53.07 ± 0.23 b	29.90 ± 0.15 g	14.84 ± 0.01 g	54.48 ± 0.17 b	30.68 ± 0.17 g
*Lactarius hepaticus*	10.00 ± 0.23 d	5.82 ± 0.08 b	31.24 ± 0.35 c	44.99 ± 0.78 e	21.24 ± 0.08 c	32.34 ± 0.38 d	46.42 ± 0.46 e
*Lentinus tigrinus*	12.25 ± 0.30 c	1.57 ± 0.10 g	28.85 ± 0.81 d	46.89 ± 0.41 e	17.16 ± 0.23 f	34.06 ± 0.69 d	48.78 ± 0.46 e
*Piptoporus betulinis*	11.96 ± 0.01 c	6.17 ± 0.30 a	8.67 ± 0.05 g	60.94 ± 0.39 c	29.10 ± 0.27 b	8.92 ± 0.06 f	61.98 ± 0.33 c
*Pluteus murinus*	6.96 ± 0.34 e	3.96 ± 0.16 d	51.53 ± 0.05 b	34.74 ± 0.39 f	13.18 ± 0.39 i	51.84 ± 0.01 c	34.98 ± 0.39 f
*Russula emética*	6.91 ± 0.35 e	5.02 ± 0.05 c	65.44 ± 0.58 a	18.47 ± 0.02 h	14.19 ± 0.58 h	65.90 ± 0.56 a	19.91 ± 0.02 h

Palmitic acid (C16:0); Stearic acid (C18:0); Oleic acid (C18:1n9c); Linoleic acid (C18:2n6c); SFA- saturated fatty acids; MUFA- monounsaturated fatty acids; PUFA- polyunsaturated fatty acids. The results are expressed in percentage. The difference to 100% corresponds to other less abundant fatty acids (data not shown). In each column different letters mean significant differences between species (*p* < 0.05).

**Table 3:** Biomolecules (vitamins and carotenoids) with antioxidant value.

Species	α-tocopherol	β-tocopherol	γ-tocopherol	δ-tocopherol	Total tocopherols (µg/100 g dw)	Ascorbic acid (mg/100 g dw)	β-carotene (mg/100 g dw)	Lycopene (mg/100 g dw)
*Amanita porphyria*	8.84 ± 0.10 c	nd	91.89 ± 11.92 c	144.85 ± 15.47 a	245.58 ± 27.29 c	211.17 ± 30.08 d	0.13 ± 0.00 d	0.01 ± 0.00 e
*Collybia fusipes*	7.75 ± 0.72 c	nd	nd	nd	7.75 ± 0.72 f	278.15 ± 12.70 a	0.24 ± 0.00 a	nd
*Fomitopsis pinicola*	5.40 ± 0.28 dce	15.50 ± 3.54 c	104.35 ± 6.86 c	nd	125.25 ± 10.13 ed	108.97 ± 2.24 g	0.22 ± 0.00 b	nd
*Hebeloma sinapizans*	2.70 ± 0.14 e	6.60 ± 0.57 c	32.51 ± 3.82 de	29.81 ± 0.00 c	71.62 ± 3.11 ef	280.55 ± 7.46 a	0.01 ± 0.00 i	0.06 ± 0.00 c
*Inocybe splendens*	1.60 ± 0.28 e	10.10 ± 0.72 c	30.90 ± 6.40 de	10.20 ± 2.25 e	52.80 ± 4.59 f	261.08 ± 3.42 bc	0.05 ± 0.00 g	0.03 ± 0.00 d
*Lactarius hepaticus*	3.88 ± 0.41 de	8.96 ± 0.88 c	3.88 ± 0.69 e	13.84 ± 2.45 d	30.56 ± 2.22 f	149.07 ± 1.76 f	nd	0.19 ± 0.00 b
*Lentinus tigrinus*	17.69 ± 1.56 b	38.98 ± 7.90 b	660.10± 72.88 a	nd	716.77 ± 79.22 a	248.13 ± 1.34 c	0.15 ± 0.00 c	nd
*Piptoporus betulinis*	3.76 ± 0.08 de	301.91 ± 27.98 a	254.25 ± 25.07 b	17.70 ± 0.02 d	577.62 ± 52.95 b	87.9 ± 3.09 h	0.09 ± 0.00 f	0.23 ±0.00 a
*Pluteus murinus*	30.02 ± 5.54 a	17.30 ± 3.34 c	62.76 ± 7.74 dc	32.77 ± 4.94 c	142.85 ± 14.88 d	173.80 ±0.73 e	0.03 ± 0.00 h	0.01 ± 0.00 e
*Russula emetica*	7.41 ± 0.83 dc	nd	16.91 ± 3.53 de	98.65 ± 5.32 b	122.97 ± 9.68 ed	266.15 ± 4.33 ba	0.11 ± 0.00 e	nd

nd- not detected. In each column different letters mean significant differences between species (*p* < 0.05).

### 2.2. Biomolecules with Antioxidant Value

**Figure 2:**
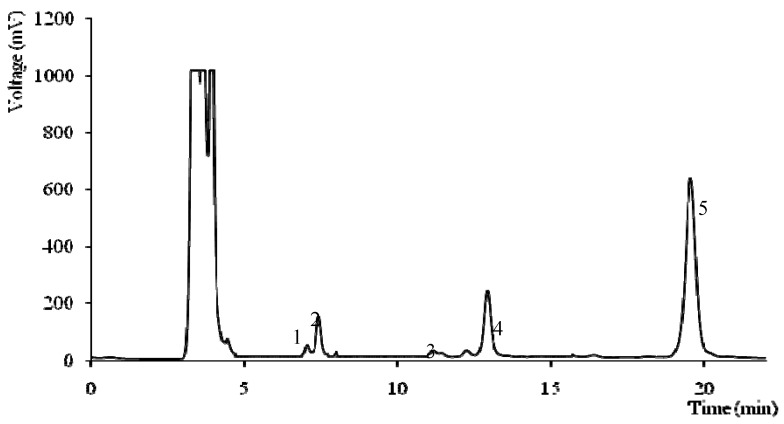
Individual tocopherols chromatogram of *Lentinus tigrinus*: 1-α-tocopherol; 2-BHT; 3-β-tocopherol; 4-γ-tocopherol; 5-tocol (IS).

Biomolecules with antioxidant properties such as vitamins and carotenoids were quantified and the results are given in Table 3. Ascorbic acid was more abundant than tocopherols. *Collybia fusipes* and *Hebeloma sinapizans* had the highest ascorbic acid content (278 and 280 mg/100 g dw, respectively). *Lentinus tigrinus* presented the highest content of tocopherols (717 μg/100 g dw) with the highest levels of γ-isoform (660 μg/100 g dw) and its profile is given in Figure 2.

Carotenoids were found in low amounts; the highest levels of β-carotene and lycopene were observed in *Collybia fusipes* and *Piptoporus betulinis*, respectively (≤0.24 mg/100 g dw). The composition in phenolics, flavonoids and *in vitro* antioxidant activity of the studied wild mushrooms is shown in Table 4. 

All the species demonstrated capacity to scavenge free radicals such as DPPH, high reducing power and capacity to inhibit lipid peroxidation in a β-carotene-linoleate system, after neutralization of the linoleate-free radical and other free radicals formed in the system which attack the highly unsaturated β-carotene models. *Fomitopsis pinicola* gave the best results in the three antioxidant activity assays, with EC_50_ values ≤ 0.13 mg/mL, which is in agreement with its highest levels of phenolics (388 mg GAE/g extract). Moreover, the antioxidant potential of this species is very promising because its reducing power EC_50_ value (0.06 mg/mL) was similar to the one obtained for the standard Trolox^®^ (0.03 mg/mL). 

The studied wild mushrooms have important antioxidant molecules (e.g. tocopherols, ascorbic acid, carotenoids and phenolics) that could play a protective role in diseases related to oxidative stress, such as cancer and cardiovascular diseases [7,9]. As far as we know, the antioxidant potential of the studied species was not previously reported, and particularly *Fomitopsis pinicola* revealed a very high phenolics concentration and powerful antioxidant properties. 

**Table 4:** Biomolecules (phenolics and flavonoids) with antioxidant value and antioxidant activity EC_50_ values.

Species	Phenolics ^a^	Flavonoids ^b^	DPPH scavenging activity ^c^	Reducing power ^c^	β-carotene bleaching inhibition ^c^
*Amanita porphyria*	57.76 ± 2.27 c	11.46 ± 0.48a	6.24 ± 0.13 d	1.28 ± 0.01 d	0.49 ± 0.07 f
*Collybia fusipes*	20.44 ± 3.23 f	4.82 ± 0.13 d	15.46 ± 0.39 b	2.22 ± 0.08 b	2.77 ± 0.00 d
*Fomitopsis pinicola*	387.70 ± 0.10 a	4.82 ± 0.31 d	0.13 ± 0.01 e	0.06 ± 0.01 f	0.10 ± 0.01 f
*Hebeloma sinapizans*	39.27 ± 1.81 d	4.82 ± 0.31 d	6.96 ± 0.32 d	1.42 ± 0.03 d	1.60 ± 0.03 e
*Inocybe splendens*	10.60 ± 1.17 h	1.50 ± 0.18 f	19.52 ± 0.82 a	2.43 ± 0.12 b	5.30 ± 0.05 b
*Lactarius hepaticus*	20.55 ± 0.81 f	7.07 ± 0.59 b	13.28 ± 0.24 b	2.16 ± 0.00 b	2.56 ± 0.39 d
*Lentinus tigrinus*	17.30 ± 1.17 g	3.77 ± 0.25 e	18.50 ± 0.52 a	2.23 ± 0.35 b	4.95 ± 0.42 c
*Piptoporus betulinis*	34.94 ± 0.01 e	6.79 ± 0.03 b	8.97 ± 0.09 dc	1.64 ± 0.01 c	1.97 ± 0.17 e
*Pluteus murinus*	9.62 ± 0.28 h	1.73 ± 0.11 f	20.62 ± 5.54 a	15.52 ± 0.67 a	5.93 ± 0.39 a
*Russula emética*	73.18 ± 0.18 b	5.82 ± 0.57 c	1.13 ±0.01 e	0.69 ± 0.00 e	0.27 ± 0.01 f

^a^ (mg GAE/g extract); ^b^ (mg CE/g extract); ^c^ (mg/mL); nd- not detected. In each column different letters mean significant differences between species (*p* < 0.05). EC_50_ values for the standard Trolox®: DPPH scavenging activity: 0.04 mg/mL; Reducing power 0.03 mg/mL; β-carotene bleaching inhibition: 0.003 mg/mL.

## 3. Experimental

### 3.1. Samples

Ten wild inedible mushrooms species were chosen in order to include different trophisms (mycorrhizal, saprotrophic and parasite) and collection habitats (*Quercus* sp., *Pinus* sp. and mixed stands). Immature and mature fruiting bodies of each species were collected in Bragança (Northeast Portugal) and the information about them is provided in Table 5.

**Table 5:** Information about the wild species analysed.

Scientific name	English name	Habitat	Ecology	Date of collection
*Amanita porphyria* (Alb. & Schwein. ex Fr.) Secr.	Grey veiled amanita	Mixed stands	Mycorrhizal	October 2009
*Boletus citrinoporus* Halling	Unknown	*Quercus* sp.	Mycorrhizal	October 2010
*Collybia fusipes* (Bull.) Quél.	Unknown	*Quercus* sp.	Saprotrophic	October 2010
*Fomitopsis pinicola* (Sw.:Fr.) P. Karst	Red banded polypore	*Quercus* sp.	Saprotrophic	October 2010
*Hebeloma sinapizans* (Paul. ex Fr.) Gillet	Unknown	*Pinus* sp.	Mycorrhizal	November 2010
*Inocybe splendens* (Heim.)	None	Mixed stands	Saprotrophic	November 2010
*Lactarius hepaticus* Fr.	Unknown	*Pinus* sp.	Mycorrhizal	November 2010
*Lentinus tigrinus* (Bull.) Fr.	Tiger sawgill	*Quercus* sp.	Saprotrophic	November 2010
*Piptoporus betulinus* (Bull. ex Fr.) P. Karst.	Birch polypore	Mixed stands	Parasite	November 2009
*Pluteus murinus* Bres.	Unknown	Mixed stands	Saprotrofic	October 2009
*Russula emetica* (Schaeff.: Fr.) Pers.	Sickener	Mixed stands	Mycorrhizal	October 2010

The identification of sporocarps was made according to several authors [15,16,17] and representative voucher specimens were deposited at the herbarium of School of Agriculture of Polytechnic Institute of Bragança. All the species were lyophilised (Ly-8-FM-ULE, Snijders, Holland) and reduced to a fine (20 mesh) dried powder; for each species, immature and mature fruiting bodies were mixed in order to eliminate the effects of maturity stage. The samples were kept at −20 °C until further analysis.

### 3.2. Standards and Reagents

Acetonitrile 99.9%, *n*-hexane 95% and ethyl acetate 99.8% were of HPLC grade from Fisher Scientific (Lisbon, Portugal). The fatty acids methyl ester (FAME) reference standard mixture 37 (standard 47885-U) was purchased from Sigma (St. Louis, MO, USA), as as were other individual fatty acid isomers, L-ascorbic acid, tocopherols (α-, β-, γ-, and δ-isoforms), sugars [D(-)-fructose, D(-)-mannitol, D(+)-raffinose pentahydrate, and D(+)-trehalose], Trolox^®^ (6-hydroxy-2,5,7,8-tetramethyl-chroman-2-carboxylic acid), gallic acid and (+)-catechin standards. Racemic tocol, 50 mg/mL, was purchased from Matreya (Pleasant Gap, PA, USA). 2,2-Diphenyl-1-picrylhydrazyl (DPPH) was obtained from Alfa Aesar (Ward Hill, MA, USA). All other chemicals and solvents were of analytical grade and purchased from common sources. Water was treated in a Milli-Q water purification system (TGI Pure Water Systems, North Andover, MA, USA). 

### 3.3. Biomolecules with Energetic Value

#### 3.3.1. Sugars

Free sugars were determined by high performance liquid chromatography coupled to a refraction index detector (HPLC-RI) as described by Grangeia *et al.* [11], using raffinose as internal standard (IS). The equipment consisted of an integrated system with a pump (Knauer, Smartline system 1000), degasser system (Smartline manager 5000), auto-sampler (AS-2057 Jasco) and a RI detector (Knauer Smartline 2300). Data were analysed using Clarity 2.4 Software (DataApex). The chromatographic separation was achieved with a Eurospher 100-5 NH_2 _column (4.6 × 250 mm, 5 mm, Knauer) operating at 30 °C (7971 R Grace oven). The mobile phase was acetonitrile/deionized water, 70:30 (v/v) at a flow rate of 1 mL/min. The compounds were identified by chromatographic comparisons with authentic standards. Quantification was performed using the internal standard method and sugar contents were further expressed in g per 100 g of dry weight (dw).

#### 3.3.2. Fatty acids

Fatty acids were determined by gas-liquid chromatography with flame ionization detection (GC-FID)/capillary column as previously described by Grangeia *et al.* [11]. The analysis was carried out with a DANI model GC 1000 instrument equipped with a split/splitless injector, a flame ionization detector (FID at 260 °C) and a Macherey-Nagel column (30 m × 0.32 mm ID × 0.25 µm *d_f_*). The oven temperature program was as follows: the initial temperature of the column was 50 °C, held for 2 min, then a 30 °C/min ramp to 125 °C, 5 °C/min ramp to 160 °C, 20 °C/min ramp to 180 °C, 3 °C/min ramp to 200 °C, 20 °C/min ramp to 220 °C and held for 15 min. The carrier gas (hydrogen) flow-rate was 4.0 mL/min (0.61 bar), measured at 50 °C. Split injection (1:40) was carried out at 250 °C. Fatty acid identification was made by comparing the relative retention times of FAME peaks from samples with standards. The results were recorded and processed using CSW 1.7 software (DataApex 1.7) and expressed in relative percentage of each fatty acid. 

### 3.4. Biomolecules with Antioxidant Value

#### 3.4.1. Tocopherols

Tocopherol content was determined following the procedure of Heleno *et al.* [10], with tocol as IS. The analysis was carried out in the HPLC system described above connected to a fluorescence detector (FP-2020; Jasco) programmed for excitation at 290 nm and emission at 330 nm. The chromatographicseparation was achieved with a Polyamide II normal-phase column (250 × 4.6 mm; YMC Waters) operating at 30 °C. The mobile phase was a mixture of *n*-hexane/ethyl acetate (70:30, v/v) at a flow rate of 1 mL/min. The compounds were identified by chromatographic comparisons with authentic standards. Quantification was based on the fluorescence signal response, using the IS method, and tocopherol contents were further expressed in μg per 100 g of dry weight (dw).

#### 3.4.2. Ascorbic acid

Ascorbic acid was determined following a procedure previously described [11] with 2,6-dichloro-indophenol, and measuring the absorbance at 515 nm (spectrophotometer AnalytikJena). Content of ascorbic acid was calculated on the basis of the calibration curve of authentic L-ascorbic acid (6 × 10^−3^–0.1 mg/mL), and the results were expressed as mg of ascorbic acid per 100 g of dry weight (dw).

#### 3.4.3. Carotenoids

β-Carotene and lycopene were determined following a procedure previously described [11] measuring the absorbance at 453, 505, 645, and 663 nm. Contents were calculated according to the following equations: β-carotene (mg/100 mL) = 0.216 × A_663_ − 1.220 × A_645_ − 0.304 × A_505_ + 0.452 × A_453_; lycopene (mg/100 mL) = − 0.0458 × A_663_ + 0.204 × A_645_ − 0.304 × A_505_ + 0.452 × A_453_, and further expressed in mg per 100 g of dry weight (dw).

### 3.5. *In Vitro* Antioxidant Properties

#### 3.5.1. Extraction procedure

Fine dried powder (20 mesh; ~1.5 g) was stirred at 25 °C with methanol (30 mL) at 150 rpm for 1 h and filtered through Whatman No. 4 paper. The residue was then extracted with one additional 30 mL portion of methanol. The combined methanolic extracts were evaporated at 35 °C under reduced pressure (Büchi R-210 rotary evaporator), re-dissolved in methanol at 20 mg/mL, and stored at 4 °C for further use. 

#### 3.5.2. Phenolics

Phenolics were determined by Folin-Ciocalteu assay. The extract solution (1 mL) was mixed with Folin-Ciocalteu reagent (5 mL, previously diluted with water 1:10, v/v) and sodium carbonate (75 g/L, 4 mL). The tubes were vortexed for 15 s and allowed to stand for 30 min at 40 °C for colour development. Absorbance was then measured at 765 nm. Gallic acid was used to obtain the standard curve (9.4 × 10^−3^–0.15 mg/mL), and the results were expressed as mg of gallic acid equivalents (GAE) per g of extract. 

#### 3.5.3. Flavonoids

For flavonoid quantification, the extract solution (0.5 mL) was mixed with distilled water (2 mL) and NaNO_2_ solution (5%, 0.15 mL). After 6 min, AlCl_3_ solution (10%, 0.15 mL) was added and allowed to stand further 6 min. NaOH solution (4%, 2 mL) was added to the mixture, followed by distilled water to a final volume of 5 mL. The mixture was properly mixed and allowed to stand for 15 min. The intensity of pink colour was measured at 510 nm. (+)-Catechin was used to calculate the standard curve (1.5 × 10^−2^–1.0 mM) and the results were expressed as mg of (+)-catechin equivalents (CE) per g of extract.

#### 3.5.4. DPPH radical-scavenging activity

This assay was performed using an ELX800 Microplate Reader (Bio-Tek). The reaction mixture in each one of the 96-wells consisted of one of the different concentrations of the extracts (30 μL) and aqueous methanolic solution (80:20 v/v, 270 μL) containing DPPH radicals (6 × 10^−5^ mol/L). The mixture was left to stand for 60 min in the dark. The reduction of the DPPH radical was determined by measuring the absorption at 515 nm. The radical scavenging activity (RSA) was calculated as a percentage of DPPH discolouration using the equation: % RSA = [(A_DPPH_ − A_S_)/A_DPPH_] × 100, where A_S_ is the absorbance of the solution when the sample extract has been added at a particular level, and A_DPPH_ is the absorbance of the DPPH solution [12]. The extract concentration providing 50% of radicals scavenging activity (EC_50_) was calculated from the graph of RSA percentage against extract concentration. Trolox was used as standard.

#### 3.5.5. Reducing power

This test was performed using the Microplate Reader described above. Different concentrations of the extracts (0.5 mL) were mixed with sodium phosphate buffer (200 mmol/L, pH 6.6, 0.5 mL) and K_3_[Fe(CN)_6_] (1% w/v, 0.5 mL). The mixture was incubated at 50 °C for 20 min, and trichloroacetic acid (10% w/v, 0.5 mL) was added. The mixture (0.8 mL) was poured in the 48-wells, as also deionised water (0.8 mL) and ferric chloride (0.1% w/v, 0.16 mL), and the absorbance was measured at 690 nm. The extract concentration providing 0.5 of absorbance (EC_50_) was calculated from the graph of absorbance at 690 nm against extract concentration. Trolox was used as standard.

#### 3.5.6. Inhibition of β-carotene bleaching

A solution of β-carotene was prepared by dissolving β-carotene (2 mg) in chloroform (10 mL). Two millilitres of this solution were pipetted into a round-bottom flask. After the chloroform was removed at 40 °C under vacuum, linoleic acid (40 mg), Tween 80 emulsifier (400 mg), and distilled water (100 mL) were added to the flask with vigorous shaking. Aliquots (4.8 mL) of this emulsion were transferred into different test tubes containing different concentrations of the extracts (0.2 mL). The tubes were shaken and incubated at 50 °C in a water bath. As soon as the emulsion was added to each tube, the zero time absorbance was measured at 470 nm. β-Carotene bleaching inhibition was calculated using the following equation: (β-carotene content after 2 h of assay/initial β-carotene content) × 100. The extract concentration providing 50% antioxidant activity (EC_50_) was calculated by interpolation from the graph of β-carotene bleaching inhibition percentage against extract concentration. Trolox^®^ was used as standard. 

### 3.6. Statistical Analysis

For each one of the species three samples were used and all the assays were carried out in triplicate. The results are expressed as mean values and standard deviation (SD). The results were analyzed using one-way analysis of variance (ANOVA) followed by Tukey’s HSD Test with α = 0.05. This treatment was carried out using SPSS v. 16.0 program. 

## 4. Conclusions

The profile of biomolecules with energetic and antioxidant potential obtained for the studied inedible wild mushrooms was similar to that of edible species [10,11,12,14]. The same energetic biomolecules were found, including the disaccharide trehalose, the monosaccharide alcohol derivative mannitol and the fatty acids palmitic, oleic and linoleic acids. The order for antioxidants abundance found in inedible wild mushrooms was: phenolics > flavonoids > ascorbic acid > tocopherols > carotenoids, similarly to that seen in edible species. *Fomitopsis pinicola* revealed a very high phenolics concentration and powerful antioxidant properties, mainly reducing power. It could find applications in the prevention of free radical-related diseases. Furthermore, this study contributes to the chemical characterization of wild species, increasing the number of documented mushroom species.
